# Nanostructured Lipid Carriers for the Formulation of Topical Anti-Inflammatory Nanomedicines Based on Natural Substances

**DOI:** 10.3390/pharmaceutics13091454

**Published:** 2021-09-13

**Authors:** Kézia Cristine Barbosa Ferreira, Ana Beatriz Caribé dos Santos Valle, Camila Quinetti Paes, Guilherme Diniz Tavares, Frederico Pittella

**Affiliations:** 1 Programa de Pós-Graduação em Ciências Farmacêuticas, Universidade Federal de Juiz de Fora, Juiz de Fora 36036-900, Brazil; keziacristine@hotmail.com (K.C.B.F.); guilherme.tavares@farmacia.ufjf.br (G.D.T.); 2 Programa de Pós-Graduação em Ciências Biológicas, Universidade Federal de Juiz de Fora, Juiz de Fora 36036-900, Brazil; abcsvalle1@gmail.com; 3 Programa de Pós-Graduação em Enfermagem, Universidade Federal de Juiz de Fora, Juiz de Fora 36036-900, Brazil; camilaquinetti@gmail.com

**Keywords:** natural products, anti-inflammatory substances, topical nanomedicines, nanostructured lipid carriers

## Abstract

The main function of the skin is to protect the body from the external environment. However, the skin can undergo inflammatory processes, due to genetic, hormonal, or environmental factors. When the defense system is overloaded, there is an increase in pro-inflammatory mediators and reactive oxygen species (ROS), which results in skin disorders. Among the substances used to treat these inflammatory processes, many natural substances with anti-inflammatory and antioxidant properties are being studied: nature is yet an abundant source to obtain diverse pharmacological actives. The treatment of skin diseases is usually focused on topical application, as it reduces the risk of systemic side effects and prevents drug degradation by first-pass metabolism. Thus, the properties of drug delivery vehicles can facilitate or inhibit its permeation. Due to the hydrophobic nature of the skin, a promising strategy to improve dermal drug penetration is the use of lipid-based nanoparticles, such as nanostructured lipid carriers (NLC). Therefore, in this review, we present NLC as a tool to improve dermal administration of natural substances with anti-inflammatory properties.

## 1. Introduction

The first barrier for the entrance of substances into our body is the skin. It is considered the first line of defense while being the largest organ of the body. This organ is responsible for protecting the underlying tissue from infections, dehydration, chemical, and physical stress and is also responsible for the wound healing process after injury [1,2,3,4]. However, genetic, hormonal or environmental factors can trigger inflammatory diseases of the skin. An abnormal immune response usually progresses on to autoimmune diseases that attack their own cells, such as psoriasis [5]. On the other hand, hormonal activity during puberty or pregnancy, for example, leads to a higher production of suet, which can cause acne. Finally, various types of substances [6,7] or microorganisms [8,9] can cause skin irritations that lead to an inflammatory process.

During the inflammatory process, pro-inflammatory mediators, such as cytokines and chemokines, attract immune system phagocytic cells to stop the inflammation onset [10]. Activated leukocytes release reactive oxygen species (ROS), aiming to kill or destroy microorganisms or degrade damaged tissue. However, non-specific targeting of ROS can cause oxidative stress to the local and adjacent cells, leading to the enhancement of the inflammatory process [10,11,12]. A well-organized system of chemical and enzymatic antioxidants protects the skin against oxidant species, avoiding deleterious effects [11,13,14]. On the other hand, this defense has limited capacity and its overload leads to an increase in ROS that results in the development of dermatological diseases [11].

Several active pharmaceutical ingredients are used in the treatment of inflammatory skin diseases, such as those with anti-inflammatory and antioxidant properties. Traditional medications are usually based on synthetic substances to treat diseases. However, in recent years, products of natural sources have gained more attention, claiming minimal side, effects compared to synthetic medicines [15,16,17,18]. Due to the high diversity of compounds produced by plants and microorganisms, research groups are screening the application of natural substances for the treatment of many diseases, including dermatological pathologies [19,20,21].

The treatment of skin diseases is mostly made by topical application since it (i) reduces the risk of systemic side effects, (ii) the drug remains concentrated in the targeted tissue, and (iii) it does not pass through the first-pass metabolism. However, the hydrophobic nature of the skin and the tightly packaging *stratum corneum* that protect the organism from the penetration of toxic agents also prevents the penetration of active substances [22,23,24]. Thus, the effectiveness of skin treatments depends on the ability of the drug to overcome the skin barrier and permeate through the epidermis [2,25]. Therefore, studies focusing on the development of nanovehicles as a predominant strategy of improving dermal penetration of drugs have emerged [18,22,24,26,27].

**Figure 1:**
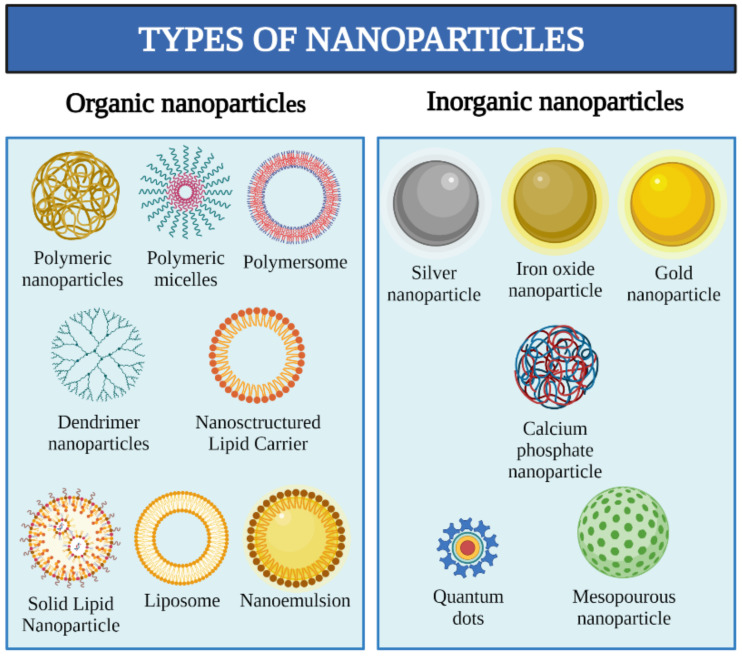
Types of nanoparticles according to their main constituent.

There have been a significant variety of nanosystems developed over the last century. Nanomaterials are frequently classified as organic and inorganic. Usually, these nanomaterials are combined to obtain improved formulation for targeted drug delivery. Figure 1 shows some of the established nanoparticles classified according to their main constituent [28]. Despite the type of material, there are general advantages regarding the nanoencapsulation of substances: (i) protection against degradation, (ii) avoidance of reticuloendothelial system activation, (iii) enhancement of bioavailability and circulation time, (iv) targeted delivery, and (v) drug solubility improvement, among others [29].

Regarding the topical delivery of natural substances, the most used nanodevices are lipid-based nanocarriers, polymeric nanoparticles, surfactant-based nanosystems, and metal-based nanoparticles, especially those prepared with gold and silver nanomaterials [30,31,32]. However, the barrier formed by the *stratum corneum* (SC), also known as the horny layer, is more effective in hindering the permeation of hydrophilic materials than lipophilic ones. Thus, the chemical nature of the delivery vehicle can facilitate or inhibit its permeation [22]. Here, we will focus on nanostructured lipid carriers (NLC). Among the advantages of this nanosystem, they present (i) high encapsulation efficiency of hydrophobic-nature substances, (ii) biocompatibility, (iii) non-toxicity, (iv) easy industrial production, and a (v) control release profile [33]. Other than that, NLC are a promising system due to the capacity to exchange lipids with the outermost layers of the SC, after skin surface binding [22,23,24,26,34]. Thus, in this review, we present NLC as a tool to improve the topical application of natural substances with anti-inflammatory properties for the treatment of skin diseases.

## 2. The Skin

The skin is the largest organ of the human body and accounts for approximately 16% of total body weight. Its vital function is to protect the body from the external environment [11,35,36,37,38]. In addition, it has important immunological and sensory functions, promotes protection against ultraviolet radiation, and helps the regulation of body temperature and the synthesis of vitamin D [36,38].

**Figure 2:**
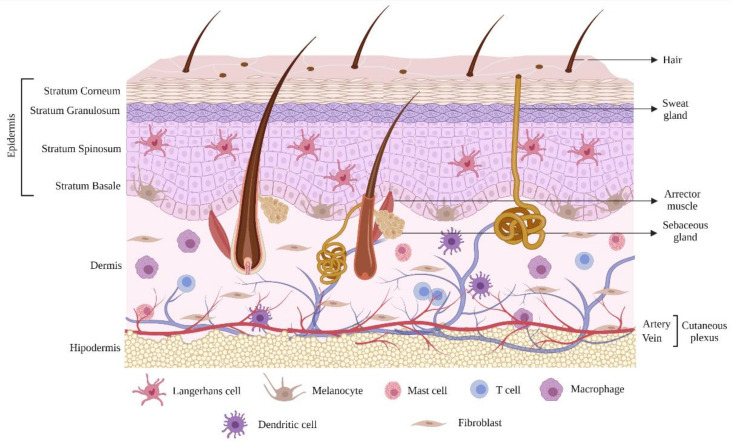
Skin structure and immune cell composition.

Anatomically it is divided into the epidermis and dermis, external and internal layers, respectively [25]. The epidermis has four sub-layers. The *stratum corneum* (SC) is the outermost sublayer, and it protects the subsequent structures of the skin, providing the primary barrier against water loss and percutaneous absorption of compounds [4,39,40,41,42,43]. The other three sub-layers of the epidermis are called the *stratum basale, spinosum,* and *granulosum* (Figure 2). Together, these sub-layers form the viable epidermis, responsible for the synthesis of the SC [4,38,39]. In some parts of the body, such as the palms of the hand and soles of the feet, there is a fifth layer. It is called the *stratum lucidum*, a dead keratinocytes layer that is located just below the SC [44,45]. This layer is responsible for the capability of the skin to stretch and lowers the effects of friction in the skin [45]. The most abundant cells of the epidermis are keratinocytes; however, other cell types are present, such as melanocytes in the *stratum basale* (responsible for the synthesis of melanin), and Langerhans cells in the *stratum spinosum* (responsible for communicating to the immune system about the presence of any foreign body) (Figure 2) [46].

The dermis is located just below the epidermis, and it is responsible for providing mechanical support and elasticity for the skin through collagen and elastin, which are produced by fibroblasts. It is highly vascularized and innervated and contains large amounts of lymphatic vessels. The epidermis appendages are found in this layer, such as hair follicles, sebaceous glands, and sweat glands. The cells that make up the dermis are fibroblasts and myofibroblasts, in addition to immune system cells (mast cell, T cell, dendritic cell, and macrophage) that offer protection against pathogens and toxic substances (Figure 2) [35,40,46,47,48].

The epidermis is in constant renewal. The keratinized cells of SC are replaced by keratinocytes from the inferior epidermis [3,40,49]. Keratinocytes migrate along with the viable epidermis and, upon leaving the basal layer, begin to differentiate both in structure and composition. During their maturation, they express and synthesize numerous structural proteins and lipids. It is at the interface between the *stratum granulosum* and the SC that the final differentiation occurs, and the keratinocytes undergo profound changes in their structure, becoming dead keratin-filled cells called corneocytes [40].

In the *stratum granulosum* or granular layer, two types of granules are formed within its cells: protein-filled keratohyalin granules and lipid-containing lamellar bodies. Following the process, in the corneal horny layer, the cell nucleus is digested, and the cytoplasm disappears. Then, lipids are released into the extracellular space, keratin intermediate filaments aggregate to form microfibrils, and the cell membrane is replaced by a cell envelope made of cross-linked protein with covalently bound lipids [38]. Finally, this novel hierarchical structure composed of layers of corneocytes embedded in a lipid-rich matrix constitutes the following layer: the SC [50]. The lipid composition of this layer is variable and presents ceramides, cholesterol, and fatty acids that are assembled into multi-lamellar bilayers [35,39,50,51]. The lipid regions of the SC form a single continuous structure. In this way, any substance that encounters the skin needs to cross these regions, which makes the organization of such lipids extremely important as an obstacle to permeation [40]. This protection against external bodies also limits drugs permeation for skin inflammation treatment.

## 3. Inflammatory Process of the Skin

The inflammatory process of the skin is characterized by pain, redness, swelling, heat, and loss of function [12,52]. In a wound-healing process, inflammation is very important for the detection and elimination of pathogenic microorganisms, removal of damaged tissue, and cleaning of the affected area [53]. The skin can suffer various types of inflammatory processes that can be caused by a series of chemical or mechanical harmful agents, pathogens, or an autoimmune or allergic response [10,52]. The inflammatory skin process is carefully detailed in Refs. [10,47,48].

**Figure 3:**
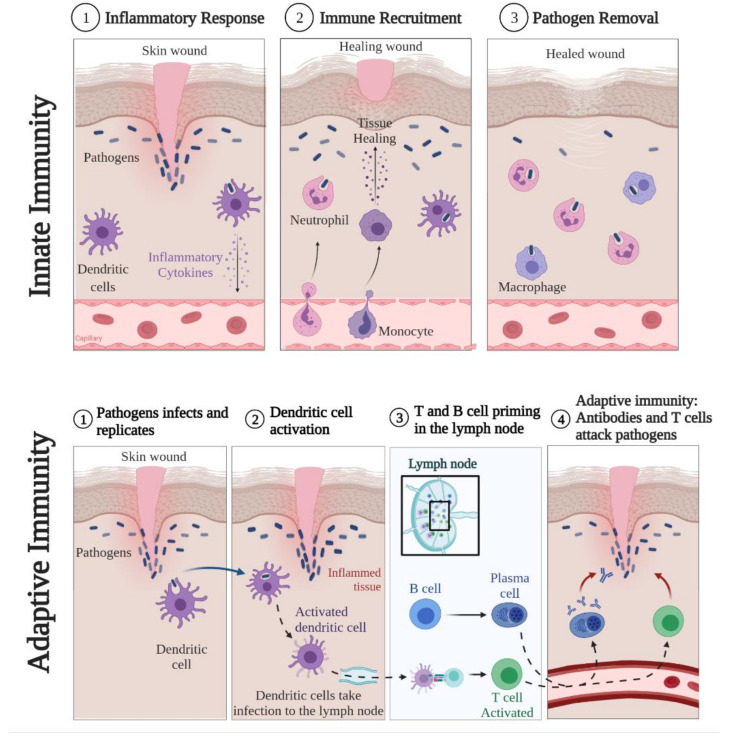
The general inflammatory process of the skin.

Briefly, after the skin barrier is disrupted, a rapid but non-specific innate immune response occurs [10,47]. In this process, the cells of the innate immune system recognize danger-associated molecular patterns (DAMPs—endogenous molecules produced or released in response to cell damage), or pathogen-associated molecular patterns (PAMPs—specific molecular structures of microbial pathogens), through receptors called pattern-recognition receptors (PRRs) [10,48]. The innate immune response leads to death and phagocytosis of the invader, and it can limit further damage and allow tissue repair [12]. The main cells that initiate this process are macrophages and dendritic cells (DC). Thus, if a DC phagocytoses a pathogen in the presence of PAMP, it will produce pro-inflammatory mediators responsible for recruiting neutrophils, monocytes, and NK cells for the initial innate immune response [10]. However, if during an infection there is resistance to this type of immunity, the body makes use of the adaptive immune response, which is slower and more specific [12]. In the case of an adaptive immune response, the process begins with the presentation of the antigen to T cells by the antigen-presenting cells (APCs). Thus, DC will migrate to the nearest lymph nodes to find the pathogen-specific T cell and recruit it to the inflammation site [10,47,48]. Finally, activated B cells secrete antibodies that reach the skin and contribute to the immune response [10] (Figure 3).

**Figure 4:**
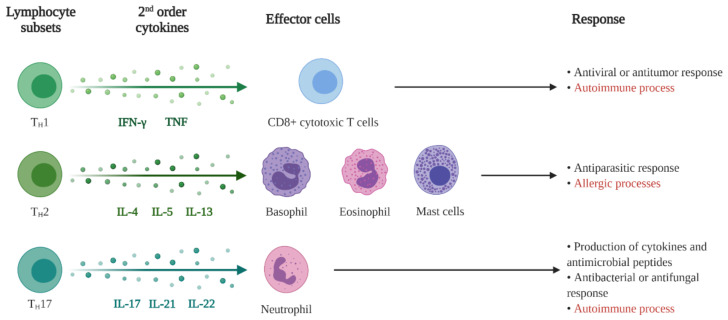
Lymphocytes activation through different signals.

Each type of signal leads to the activation of a different subgroup of Th cells (Figure 4). Thus, Th1 cells are activated after a viral infection or tumor cells and produce interferon-γ (IFN-γ) and tumor necrosis factor (TNF), to recruit CD8 + cytotoxic T cells (CTLs) for an antiviral or antitumor response. Th2 cells, on the other hand, respond to parasites and produce IL-4, IL-5, and IL-13 that recruit basophils, eosinophils, and mast cells to coordinate an antiparasitic response. Finally, Th17 cells respond to bacteria and fungi infections and produce IL-17, IL-21, and IL-22, which recruit neutrophils and promote an antibacterial or antifungal response [10]. In the case of skin inflammation caused by an autoimmune process, such as in vitiligo, lupus, and psoriasis, this response is promoted by Th1 and Th17 cells that lead to a misdirected response against the body itself. In allergic processes, however, the response is Th2 and occurs, for example, in allergic contact dermatitis that occurs after chemical or environmental exposure [10,48]. After the inflammatory process finally manages to control the threat, regulatory T cells suppress immune responses, helping to prevent autoimmunity and attenuate inflammation. Without this cell type, several autoimmune skin diseases could be generated, such as eczema, psoriasiform dermatitis, urticaria, and alopecia universalis [10].

In an efficient healing process, the transition between the inflammatory and proliferative phase occurs by decreasing the number of neutrophils, macrophage phenotypic, collagen deposition, and revascularization. However, during the inflammatory process, disturbances and excessive production of pro-inflammatory mediators can occur, which causes inflammation to become pathogenic and leads to the development of chronic inflammatory diseases. Interactions between skin cells, such as fibroblasts and keratinocytes, occur and impair the tissue remodeling process. Thus, chronic wounds are marked by high levels of inflammation, decreased production of growth factors and proliferation of endothelial cells, and by non-re- epithelialization [48,52,53]. It is believed that ROS are also involved in chronic inflammatory responses, after an overload of the skin’s antioxidant capacity. During an inflammatory process, ROS are important for defense against a pathogen and degradation of damaged tissue. However, due to the high reactivity of these radicals, they can oxidize molecules, such as proteins, lipids, and DNA from local and adjacent cells, causing oxidative stress and therefore increasing the inflammatory process. Oxidative stress is characterized by excess ROS or an insufficient number of antioxidants and can increase the inflammatory process by (i) incorrectly oxidizing host cell constituents that cause immune cells to respond; or (ii) activating redox-sensitive proteins, such as the transcription factor NFκB, which leads to the increased expression of pro-inflammatory mediators [11,12,13].

## 4. Natural Substances with Anti-Inflammatory Activity

Natural products coming especially from plants and fungus have been used for thousands of years to treat countless diseases [54]. Between 1981 and 2002, for example, 62% and 64% of new anticancer molecules and antihypertensive drugs, respectively, were obtained from natural sources [55]. In addition, different molecules coming from natural products can have specific activities, such as antibacterial, antifungal, antimicrobial, analgesic [56], anti-inflammatory, and antioxidant properties [57,58,59].

Among these conditions, inflammation is caused by tissue injury (e.g., stress, irritants, and radiations), infections (microbial and viral), or genetic changes that lead to a complex biological reaction induced by the disruption of the tissue homeostasis [15,16,60]. The main chemical constituents found in natural products that are known to have anti-inflammatory or antioxidant activities are listed in Table 1.

The molecules highlighted in Table 1 are originated and extracted by plants species. Other compounds with anti-inflammatory and antioxidant activities used to treat inflammatory skin diseases can also be isolated from fungi and bacteria. Cyclosporine is a lipophilic immunosuppressant extracted from fungi species that acts by blocking lymphocyte functions through calcineurin inhibition, after cyclophilin. Consequently, the production of pro-inflammatory cytokine interleukin-2, responsible for lymphocyte maturation, is compromised [87]. Like cyclosporine, tacrolimus also acts by inhibiting calcineurin. However, it binds to another immunophilin, FKBP. This connection could start the production of several inflammatory mediators. This lactone can be isolated from *Streptomyces tsukubaensis* and it has similar properties and mechanisms to cyclosporine [87]. Additionally, high amounts of IL-8 are observed in psoriasis, which stimulates keratinocytes division. Therefore, an important role of tacrolimus is that it can act directly on these cells, reducing the number of IL-8 receptors, relieving inflammation [88,89].

An advantage of the use of natural products as a source of substances for the treatment of diseases is that they are usually found in high abundance in nature, which makes the substances often cheaper than their synthetic counterparts [58].

Ultimately, the treatment of skin inflammatory diseases using natural substances depends on its permeation through the SC, composed of lipid regions that form a single continuous structure. Overall, the skin has low permeability to the penetration of foreign molecules, thereby protecting the body [50]. Consequently, rational strategies must be applied to overcome the barriers of the skin [41].

## 5. Nanostructured Lipid Carriers (NLC)

Lipid-based nanoparticles are an effective strategy to improve drug absorption by skin [90,91]. Among them, solid lipid core nanoparticles (SLCN) appear to be a promising approach to improve treatment efficacy by increasing the active delivery to the epidermis [34,92,93,94]. This type of nanosystem presents a solid lipid core at room and body temperature, and is mainly divided into solid lipid nanoparticles (SLN) and nanostructured lipid carriers (NLC).

Müller and Lucks introduced the term solid lipid nanoparticle (SLN) in 1996, after developing a novel method for the production of lipid nanoparticles, using high pressure homogenization [95,96]. They are composed exclusively of solid lipids or a blend of solid lipids [97]. On the other hand, nanostructured lipid carriers (NLC) also present a solid lipid core and are prepared by a blend of solid and liquid lipids to yield a non-crystalline amorphous lipid core, which allows higher drug loading [96]. Both types of nanoparticles (SLN and NLC) are encompassed as solid lipid core nanoparticles (SLCN).

**Figure 5:**
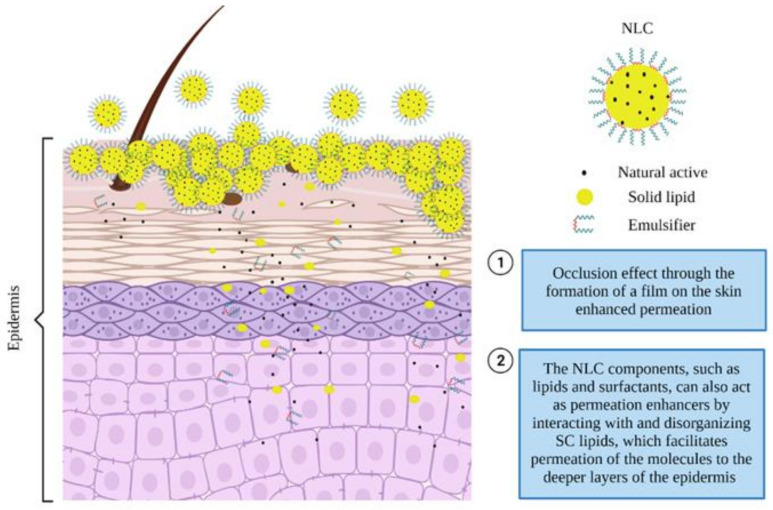
Permeation and penetration of nanostructured lipid carriers in the skin after topical administration.

Due to the similarity in lipid nature of the core of produced nanoparticles and the epidermal lipids, NLC have enhanced the permeation of drugs after topical application [93,98]. The enhanced permeation happens because NLC cause an occlusion effect through the formation of a film on the skin (Figure 5) [99,100]. The occlusive effect reduces transepidermal water loss, improving hydration of the skin, and increasing drug penetration [18,27,43,101,102,103,104]. This effect is conferred by the small size and strong adhesive properties of these particles [43,99,102,104,105]. In addition, NLC components, such as lipids and surfactants, can also act as permeation enhancers by interacting with and disorganizing SC lipids, which facilitates permeation of the molecules to the deeper layers of the epidermis [20,43].

Thus, inflammatory skin diseases can be successfully treated by the localized release of actives through NLC, more efficiently than conventional formulations. These nanosystems may improve the anti-inflammatory properties of the active by increasing its permeation through (i) targeted epidermal delivery, (ii) *stratum corneum* surface modification after contact of the nanocarrier’s components and the corneocytes, (iii) nanoparticle’s adherence to the skin surface by the occlusion effect, which leads to a controlled release of the active, and (iv) enhanced active concentration by increasing the solubility of the incorporated actives, among others [18,20,61].

Among the advantages of NLC, the lipids used in the composition of these systems (i) are usually non-irritating and non-toxic, (ii) allow the encapsulation of lipophilic compounds, (iii) increase drug stability, (iv) protect the active against degradation, (v) improve the drug load, (vi) possess eco-friendly production methods, and (vii) present ease of sterilization [106,107,108,109,110,111,112,113]. Together, these characteristics make NLC excellent vehicles for actives used in the treatment of inflammatory skin diseases.

### Constituents and Methods

**Figure 6:**
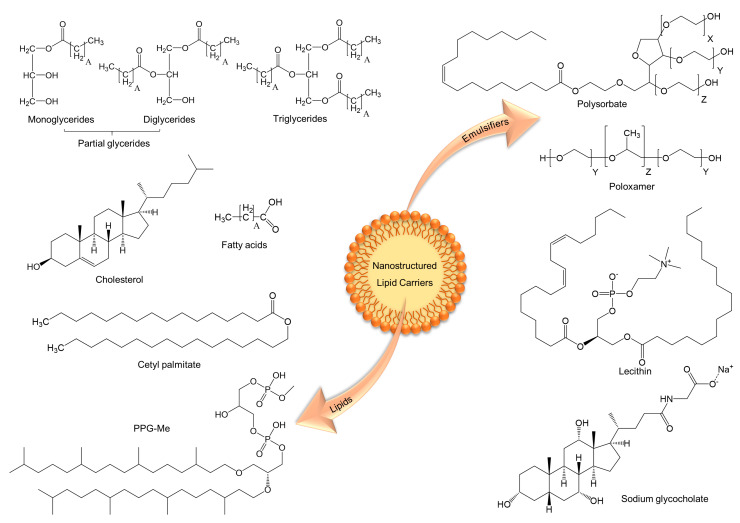
Illustration of nanostructured lipid carriers (NLC) and the main lipids and emulsifiers used for their construction.

Nanostructured lipid carriers (NLC) are formed from a blend of solid and liquid lipids, emulsifiers and water (Figure 6) [114,115,116]. Among the lipids used in the formulation, triglycerides (e.g., tristearin, tristearate, tripalmitate, tripalmitin), partial glycerides (e.g., Imwitor), fatty acids (e.g., palmitic and stearic acid), steroids (e.g., cholesterol), and waxes (e.g., cetyl palmitate) are highlighted [92,114,116,117,118]. Furthermore, recent studies report the use of archaeolipids for the construction of lipid nanoparticles [119,120]. This type of lipids is extracted from the hyperhalophilic archaeobacteria *Halorubrum tebenquichense*, and the major component is 2,3-di-O-phytanyl-sn-glycero-1-phospho-(3’-sn-glycerol-1’-methyl phosphate) (PGP-Me). Lipids that form highly crystalline particles with a perfect lattice (e.g., monoacid triglycerides) have low drug integration capacity, while more complex lipids, such as mixtures of mono-, di- and triglycerides and fatty acids of different chain lengths form less perfect crystals with many imperfections, offering space to accommodate drugs [121].

NLC formulations are compatible with most emulsifiers (e.g., poloxamer 188, polysorbate 80, lecithin, and sodium glycocholate) approved by drug regulatory agencies [118,121,122]. In addition, recent papers showed that a combination of emulsifiers can be used to prevent particle agglomeration [92,114,123].

Different methods can be used for the production of NLC, encapsulating natural substances, such as high-pressure homogenizations, ultrasound, microemulsion, solvent evaporation, spray-drying, and others, as already reviewed in detail by Dhiman and co-workers and Mishra and collaborators [33,124]. Here, we focus on the three main techniques used for natural compounds encapsulation: high-pressure homogenization, microemulsion technology, and ultrasound methods.

High pressure homogenization: it is the most used technique for the preparation of NLC. Compared to other techniques, it has the advantage of large-scale transposition, which normally presents reproducible results [100,114]. In this method, the particulate dispersion is driven with high pressure (100–2000 bar) through a narrow cavity (few micrometers) and accelerated at a very short distance with very high speed (above 1000 km/h). Shear stresses and very high cavitation forces rupture the particles causing them to assume nanoscale diameters [100,106,109,114,125].

- Ultrasound: In this method, the particles are formed by ultrasonic waves that generate cavitation in liquids. Thus, when a liquid is subjected to the process of sonication with high intensity, the sound waves propagate in the middle of the liquid, creating alternation of high- and low-pressure sound waves. In the phase of low pressure and high intensity, the waves produce vacuum bubbles, which increase the diameter by absorbing energy. After reaching the high-pressure phase, the bubbles are compressed until they implode [126,127]. By using this method, researchers have to strictly control the conditions to avoid wide particle size distribution, which leads to physical instabilities of the formulation [100,125].
- Microemulsion: This method was first used by Gasco et al. (1997) [96,109,114]. Microemulsions typically contain unsaturated fatty acids, surfactants, co-surfactants, and water. They are mixed at low-speed stirring, which forms an optically transparent mixture at 65–70 °C. The hot microemulsion is then dispersed in cold water (2–3 °C) under gentle agitation resulting in the solidification of nanostructured lipid carriers [100,109,114,121,125].

Regarding sterilization, the most used techniques include γ-radiation, autoclaving, and filtration. In addition, the sterile product can be obtained by aseptic production, using good manufacturing practice. The γ radiation should be used in systems with a low probability of chemical reactions between the components since free radicals are formed in all the samples due to the high energy of the γ rays [114]. In the case of autoclaving, the lipid melting temperature and composition of NLC are critical parameters for the applicability of this process to sterilize this type of nanoparticles. The choice of a suitable surfactant, for example, is very important for the physical stability of NLC, even at high temperatures [114,128]. Unlike the two previous forms of sterilization, filtration requires the particles to have a diameter below the pore size of the filter in use [114].

Finally, adequate characterization of NLC is necessary for its quality control [114,125]. The important parameters evaluated for the NLC include particle size, polydispersity index, surface charge (zeta potential), degree of crystallinity and lipid modification (polymorphism), the coexistence of additional colloidal structures (micelles, liposomes), encapsulation efficiency, in vitro drug release, morphology and stability [18,114,125].

## 6. NLC Containing Natural Substances against Skin Inflammation

Natural substances presented in Table 1 possess a hydrophobic nature and, as naked substances (without a delivery system), exhibit low solubility in water, chemical or photoinstability, and low bioavailability, which may impair the pharmacological effect. The administration of those substances without any protection system can be unspecific and cytotoxic, and may promote various side effects within the organism, especially if systemically administered [129]. On the other hand, after topical administration, some of them may go beyond skin layers and also reach circulation, resulting in a low skin retention time [123]. After reaching blood circulation, unprotected natural compounds may attract protein corona and activate the reticuloendothelial system, limiting bioavailability while enhancing renal clearance [130]. Thus, the use of nanovehicles, such as NLC, is being applied to improve the safety and efficiency of natural substances.

**Figure 7:**
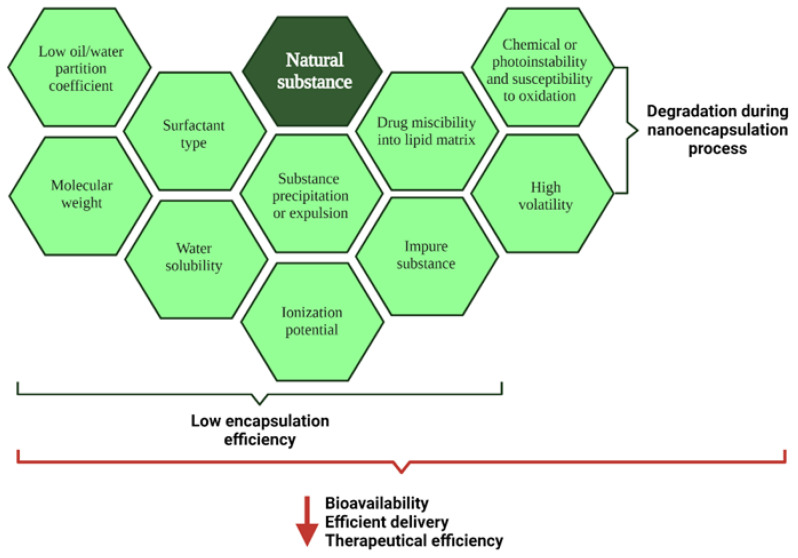
Technical challenges to encapsulate natural substances into NLC.

When preparing drug delivery systems, compounds from natural sources may face challenges during nanoencapsulation. Depending on the chemical structure, molecular weight, water affinity, and drug miscibility within the lipid matrix, the incorporation into nanostructured carriers may be compromised (Figure 7).

Lipid-based nanosystems are usually applied to encapsulate hydrophobic substances as they also exhibit hydrophobic compartments. Therefore, regarding SLN and NLC, drug miscibility in the matrix is very important. Authors have compared several types of core composition and observed differences in the encapsulation efficiency, considering the type of lipid used to synthesize the nanocarrier [17]. Additionally, the amorphous core structure of NLC is more likely to store a greater amount of the substance as observed by Puglia et al. (2017) and Mura et al. (2021) [17,131]. The ionization potential of natural substances also has to be carefully observed. This will demand pH control or the use of a counterion to ensure that the molecule will maintains its affinity to the lipid core [132]. All of the above characteristics can impair the encapsulation efficiency within the nanocarrier by substance precipitation and even expulsion of the lipid matrix to affect their therapeutical efficiency [125].

Challenges are also observed when natural substances are not well purified after extraction. The use of total extracts can affect the encapsulation efficiency of a specific substance, due to the mixture of molecules that can cause chemical incompatibility. The components from the mixture will be distributed in the external aqueous phase, adsorbed on the surface of the nanoparticle, or encapsulated in the core. It also affects the therapeutic efficiency since the absorption and release of each compound will depend on its location in the nanoparticle suspension [133]. Likewise, the choice of an appropriate surfactant will drive the absorption of the substance (thus the location in the nanoparticle) and the final release, while conferring stability to the dispersion. The choice of surfactants should be analyzed in combination with the other components of the formulation and should be kept at the lowest concentration possible, as they may be skin irritants and impact inflammatory processes [117,134].

Besides affecting the encapsulation efficiency, high volatility and molecular instability may also disturb the integrity of the compound during the nanoencapsulation process. If there is chemical or photoinstability, the product of degradation may be formed, and thus monitored, during the process. Depending on the type of method used for nanocarrier production, the molecule may volatilize, especially if heated [135]. Finally, post-manufacture steps, such as transportation and storage, have to be also carefully provided [136].

Ultimately, the use of NLC leads to the protection of unstable chemical substances and allows the controlled release in the extracellular environment or the penetration in targeted cells for intracellular delivery. Altogether, NLC enhance the therapeutic effects of the bioactive compounds derived from natural products [16,137]. In Table 2, we present studies using NLC to encapsulate natural compounds with anti-inflammatory and antioxidant activity for topical administration against skin diseases.

All the compounds that use NLC as a delivery technology are hydrophobic, so they are able to be complexed in the lipid core. The main reason to use this technology is the fact that it protects labile substances, enhances the permeability of the hydrophobic compounds, and allows sustained release after a single application [150].

Three studies analyzed curcumin incorporated into NLC and found similar results for the permeability of curcumin [26,123,143]. In a permeation study, Caon et al. (2017) observed through fluorescence microscopy that when curcumin was incorporated into NLC, the concentration of this active remained high in the superficial layers of the skin. This suggests that NLC is suitable for the topical administration of curcumin by reducing its high permeability through the skin [143]. Shrotriya and colleagues (2018) prepared a NLC containing curcumin and incorporated it into a carbopol gel. They observed that the gel containing NLC exhibited controlled drug release up to 24 h, as the permeation of the drug through the skin was lower than that of the plain gel. This indicates that curcumin was retained in the epidermis and dermis when incorporated into NLC, which was confirmed by a drug deposition study. In addition, the NLC gel showed high occlusion properties and a slight increase in antioxidant activity, compared to conventional gel [26]. In accordance, Zamarioli et al. (2015), showed that there was no permeation of curcumin in the pig ear skin for up to 18 h. This suggests that NLC modified the permeation of the curcumin and controlled its release, increasing the residence time in the superficial layers of the skin [123]. In addition, topical co-delivery of curcumin and caffeine by gel-incorporated NLC was accomplished by Iriventi and Gupta for antipsoriatic activity. In vivo studies showed promising results, as the tested formulation alleviated symptoms by day 8, while market formulation showed similar results by day 20 [144].

The carotenoids are a family of natural lipid-soluble pigments found in plants that are also known to have anti-inflammatory activity [151,152]. Fucoxanthin is a marine pigment produced by microalgae and brown macroalgae. This carotenoid has anti-inflammatory and antioxidant activities and was incorporated in NLC by Cordenonsi and co-workers (2019). Besides good physicochemical properties, the skin porcine permeation study showed specific and linear NLC distribution without transdermal delivery [61].

Other than that, Mitri and co-workers (2011) observed an increase in chemical and photostability of the carotenoid lutein, after incorporation in NLC. In addition, the NLC formulation led to a sustained release of the active and also to an increase in penetration rates, compared to free lutein [145]. Another carotenoid with anti-inflammatory and antioxidant activities is lycopene. This is a lipophilic pigment and one of the most potent antioxidants known [146]. As it is very unstable, Okonigi and Riangjanapatee (2014) prepared NLC to protect lycopene, delaying its chemical degradation. In addition, the NLC led to a biphasic release profile of lycopene, which is relatively rapid during the first 6 h, followed by a prolonged release over the next 18 h [146].

Another substance from natural sources incorporated in NLC was psoralen. Psoralen acts by binding to the DNA molecule when exposed to UV light, inhibiting its synthesis, and consequently, it decreases cell proliferation [73,153]. This substance is mainly used to treat psoriasis, a skin inflammation that is characterized by the uncontrolled cell proliferation of the skin. Psoralen was incorporated into SLN/NLC by two studies, aiming its topical use to inflammatory skin diseases, such as psoriasis [107,154]. Faiyazuddin and colleagues (2010) encapsulated babchi oil, which is mainly composed of psoralen, to SLN formulations. They found that skin permeation was improved using SLN formulations. In addition, the drug release of SLN formulations was found to be more rapid in the first 24 h, when compared to the free compound, probably due to the initial burst release [154]. Fang and co-workers (2008) carried out another study that used nanostructured psoralen. They showed that the NLC formulations enhanced permeation and controlled release of the drug. In addition, they observed that the psoralen derivative, 8-methoxypsoralen, permeated similarly in normal or hyperproliferative skin, compared to the free drug [107].

Quercetin is the flavonoid that has the highest antioxidant property. Besides this activity, it also presents other pharmacological activities, such as being anti-inflammatory [20]. In the work of Chen-Yu and co-workers (2012), NLC promoted the permeation and increased the amount of substance retained in the skin, compared to the quercetin-containing polyethylene glycol solution. In addition, NLC *per se* also enhanced the anti-oxidative and anti-inflammatory effect exerted by quercetin, which is favorable for the treatment of inflammatory conditions [20]. Another NLC-quercetin formulation was developed by Bose and Michniak-Kohn (2013) and promising results were observed regarding topical administration. Yet, in comparison to the SLN formulation, the NLC system showed the highest delivery of quercetin, revealed by the quantification of retained quercetin in the skin [147].

The natural polyphenolic compound resveratrol has a potent antioxidant with strong anti-inflammatory properties. Two studies analyzed resveratrol incorporated into NLC [113]. Sun and colleagues compared nanoemulsion, SLN, and NLC formulations encapsulating resveratrol for topical delivery [148]. Regarding the two last formulations, NLC showed a more controlled release profile than SLN. On the other hand, SLN had a better outcome concerning skin permeation. Nevertheless, NLC formulation had a great outcome concerning topical delivery of resveratrol. In accordance with these results, Soldati and collaborators (2018) found that the release profile of resveratrol from the NLC was biphasic, with a burst release within the first 4 h followed by a prolonged release over 24 h. Additionally, NLC improved the permeation and retention of the resveratrol in the upper layers of skin and enhanced by 20% the antioxidant activity of resveratrol, compared to the free active [113].

Another phenolic compound incorporated into NLC was sesamol, which has antioxidant activity [17]. Puglia and colleagues (2017) were able to control the rate of sesamol diffusion through the skin by incorporating it into an NLC/SLN, thereby maintaining high concentrations of sesamol in the upper layers of the skin. In addition, NLC and SLN prolonged the antioxidant activity of sesamol up to 40 h [17].

Terpenes are a family of organic compounds found especially in essential oils produced by plants and are not only known to be valuable penetration enhancers by causing disorders on the integrity of the *stratum corneum* structure [155], but also to have important anti-inflammatory properties [156]. In the work of Pivetta and collaborators (2018), thymol was incorporated into NLC, and a biphasic release was obtained. In addition, the NLC formulation presented better anti-inflammatory and antioxidant activity than the free thymol. Both inflammation models used to test the nanoparticles showed inhibition of 60% of the edema. This can be explained due to the high permeation capacity of this terpene, which is highly lipophilic, allowing the mobility of the *stratum corneum* lipid structure [91].

Regarding the compounds produced by fungi and bacteria, several groups are developing nanostructures to improve their anti-inflammatory activity, such as cyclosporine A (CyA) and tacrolimus. Kim and co-workers (2009) incorporated cyclosporin A into NLC and obtained high permeation of the active into the *stratum corneum* (7.4 times greater than the cyclosporin A oil mixture). This was also observed in the viable skin, where permeation was 2.4-fold greater for the nanostructured cyclosporin A. Yet, an in vivo experiment showed that cytokines levels of IL-4 and IL-5, normally overexpressed in atopic dermatitis, decreased by 40% in both cases [138]. Topical CyA delivery was also achieved by Silva and colleagues (2020) [140], Essaghraoui and co-workers (2019) [139], and Trombino and collaborators (2020) [141]. Efficient permeation was observed with reduced transdermal permeation. Thus, this indicates promising topical administration formulations by all works. In addition, co-delivery of CyA and calcipotriol by NLC was performed for psoriasis treatment. Ex vivo permeation studies reported a non-detectable quantity of either compound, which means skin retention and no transdermal delivery. Additionally, the actives penetrated deeper when encapsulated, compared to free administration. In vivo studies observed maximum reduction in skin inflammation by the nanoformulation with no scaly lesions and reduced thickness of the skin [142].

Regarding co-delivery, tacrolimus and TNF-α siRNA were co-encapsulated into a NLC by Viegas and co-workers (2020) into a multifunctional NLC. They observed good physicochemical properties and promising permeation and retention profiles for topical application. Finally, in vivo studies showed synergic effect and a more preserved architecture of the tissue after tacrolimus and TNF-α siRNA co-delivery in the psoriasis model [149].

In addition, a complex mixture was nanoencapsulated and presented improvement regarding its anti-inflammatory properties by topical administration. Afra and collaborators (2020) proposed propolis flavonoids incorporation within NLC for topical treatment. In vitro release reported an initial burst followed by a prolonged release up to 24 h post-incubation. Furthermore, ex vivo assays demonstrated higher effectiveness in penetration and permeation, and in vivo studies showed a significant reduction in the volume of the edema after NLC topical administration [157]. Another study by Lacatusu and colleagues (2017) aimed to incorporate a marigold extract and azelaic acid into SLN. The co-presence of these compounds promoted a reduction in IL-6 and IL-1β pro-inflammatory cytokines. In addition, paw edema was significantly reduced after NLC treatment [153].

As shown in Table 2 and discussed here, the nanoencapsulation of natural substances with anti-inflammatory activity into nanostructured lipid carriers for topical application is yet a broad field for the development of novel nanomedicines. The enriched natural diversity allied to recent advances in the preparation methods and nanomaterials provides an open field for the development of novel anti-inflammatory nanomedicines.

## 7. Conclusions

The treatment of topical inflammatory diseases is usually based on anti-inflammatory and antioxidant substances applied to the skin. Among the compounds, those extracted from natural sources stand out because of their effectiveness and low cost, in addition to patient compliance in the use of natural products. Topical application is prioritized due to direct action on the target tissue, which decreases the risk of systemic side effects and prevents drug degradation by first-pass metabolism. However, two main obstacles that difficult their penetration into the skin must be considered: the characteristics of the substance itself and the skin barriers. Thus, nanostructured lipid carriers appear to be a way of overcoming these problems, allowing effective targeting of the drug to the epidermis. Consequently, it increases the efficiency of the treatment and reduces systemic absorption, making this type of carrier ideal for topical application.

## Figures and Tables

**Table 1:** Chemical groups and mechanism of action of natural substances extracted from plants that present anti-inflammatory activity.

Chemical Group	Examples of Substances	Mechanism of Action as an Anti-Inflammatory
Carotenoids	Fucoxanthin	Acts by restraining tyrosinase activity [61] and nitric oxide production. It also inhibits nitric oxide synthase, cyclooxygenase 2 (COX-2), and prostaglandin protein expressions. In the same way, TNF-α, IL-1β, and IL-6 are reduced after fucoxanthin treatment [62].
	Lutein	Decreases pro-inflammatory cytokines such as IL-6, IL-1β, and TNF [63,64]. Inhibits cyclooxygenase expression [63], which downregulates the activation of prostaglandin [65]. Acts through radical scavenging activity by AP-1 pathway [63].
	Lycopene	Inhibits pro-inflammatory proteins, such as TNFα, IL-1β, IL-6, and IL-8, by the NF-kβ pathway and induces the expression of anti-inflammatory cytokines, such as IL-10 [66]. Presents antioxidant activity due to its eleven conjugated double bonds [67]. Inhibits redox by suppressing ROS-producing enzymes like cyclooxygenase, lipoxygenase, nitric oxide synthase, and NADPH oxidase [68].
Flavonoids	Quercetin	Suppresses pro-inflammatory pathways, such as AP-1, cyclooxygenase, and NF-kβ, therefore, inhibiting several pro-inflammatory cytokines, as TNF-α and IL-1β [69] and iNOS [70]. It also inhibits xanthine oxidase and lipoxygenase, decreasing oxidative injury [71], and quinone reductase 2 that catalyzes toxic compounds, forming ROS [72].
Furocoumarin	Psoralen	Inhibits cell division and proliferation through DNA interaction [73]. Decreases the levels of pro-inflammatory cytokines, such as IL-1β [74].
Phenolics	Curcumin	Produces anti-inflammatory effect through the peroxisome proliferator-activated receptor gamma (PPAR-y) pathway [75]. Causes the reduction of NF-kβ and AP-1 pathways, which inhibits pro-inflammatory mediators, such as TNF-α and other cytokines [75,76]. Blocks the formation of ROS and the production of pro-inflammatory cytokines by inhibiting cyclooxygenase [77]. Scavenges reactive species, modulates the activity of glutathione peroxidase, catalase, and superoxide dismutase, besides inhibiting ROS-generating enzymes such as cyclooxygenase, and also lipoxygenase, and xanthine hydrogenase [76].
	Resveratrol	Inhibits the AP-1 and NF-kβ pathways [78,79]. Blocks the expression of cyclooxygenase and cytokines, such as IL-1, IL-8, iNOS, and TNF-α [78,80,81,82]. Upregulates anti-inflammatory cytokines, such as IL-2 and IL-10 [83]. Scavenges the reactive oxygen species [78].
	Sesamol	Inhibits cytokine production of TNF-α and IL-1β by suppressing the NF-kB pathway [84]. Inhibits lipoxygenase through its radical scavenging activity, due to the presence of a benzodiol group [85].
Terpenoids	Thymol	Inhibits cyclooxygenase. Inactivates calcium channels by triggering the reduction of elastase [86].

**Table 2:** Topical anti-inflammatory nanomedicines based on nanostructured lipid carriers containing natural substances.

Natural Compound.	Biological Activity	Limitations	Results
Cyclosporin A [138,139,140,141,142]	Immunosuppressant	Poor water solubility. Systemic side effects after oral administration. Low permeation of cyclosporin A through the skin.	NLC permeation was higher than free cyclosporin A. No systemic absorption.
Curcumin [26,123,143,144] and curcuminoids [21,123]	Anti-inflammatory and antioxidant	Poor water solubility. High permeation through the skin, reaching blood vessels.	NLC loading curcumin was concentrated in the superficial layers of the skin, reducing the permeation of curcumin. NLC gel showed an enhanced skin drug deposition. Slight increase in antioxidant activity.
Fucoxanthin [61]	Anti-inflammatory and antioxidant	Photoinstability.	Improved photostability. Fucoxanthin protection against degradation. Assured topical administration without transdermal penetration.
Lutein [145]	Anti-inflammatory and antioxidant	Poor water solubility. Low bioavailability. Unstable molecule.	NLC increased lutein’s chemical and photostability. NLC provided a sustained release. NLC increased penetration rates.
Lycopene [146]	Antioxidant and anti-inflammatory	Poor water solubility. Low permeation of lycopene through the skin. Unstable molecule.	NLC retarded the chemical degradation. NLC led to a biphasic release profile.
Psoralen [107]	Anti-inflammatory and anti-proliferative	Poor water solubility. Photosensitive molecule.	NLC enhanced permeation through the skin.
			NLC led to a biphasic release profile. Skin permeation was improved.
Quercetin [20,147]	Anti-inflammatory and antioxidant	Poor water solubility. Low skin permeability.	NLC promoted permeation. Enhanced the effect of anti-oxidation and anti-inflammation.
Resveratrol [113,148]	Antioxidant and anti-inflammatory	Poor water solubility. Poor bioavailability. Photosensitive.	NLC enhanced the deposition of the drug in the skin. NLC controlled the release of the active. NLC enhanced the antioxidant activity. NLC improved the permeation of resveratrol.
Sesamol [17]	Anti-inflammatory and antioxidant	Poor water solubility. High permeation through the skin.	NLC controlled the rate of sesamol diffusion through the skin. NLC prolonged antioxidant activity.
Tacrolimus [149]	Immunosuppressant	Low bioavailability. Skin irritations (brun sensations, pruritus). Stickiness.	Controlled drug release followed by a sustained release. NLC did not cause erythema and edema. NLC enhanced the permeation ability of tacrolimus and dermal accumulation. The inflammatory activity was higher in NLC-tacrolimus treatment when compared to the reference. NLC formulation reduced undesirable stickiness.
Thymol [91]	Anti-inflammatory and antioxidant	Unstable molecule. Skin irritations. High permeation through the skin.	NLC improved the stability of the molecule. NLC eliminated skin irritations, such as erythema. NLC controlled the penetration of thymol through the skin. NLC provided a biphasic release profile. Consequently, NLC improved anti-inflammatory and antioxidant activity.

## References

[1] Antunes-Ricardo M., Gutiérrez-Uribe J.A., Martínez-Vitela C., Serna-Saldívar S.O. (2015). Topical anti-inflammatory effects of isorhamnetin glycosides isolated from Opuntia ficus-indica. BioMed Res. Int..

[2] Kwon S.S., Kim S.Y., Kong B.J., Kim K.J., Noh G.Y., Im N.R., Lim J.W., Ha J.H., Kim J., Park S.N. (2015). Cell penetrating peptide conjugated liposomes as transdermal delivery system of *Polygonum aviculare* L. extract. Int. J. Pharm..

[3] Fuchs E. (2007). Scratching the surface of skin development. Nature.

[4] Yagi M., Yonei Y. (2018). Glycative stress and anti-aging: 7. Glycative stress and skin aging. Glycative Stress Res..

[5] Greb J.E., Goldminz A.M., Elder J.T., Lebwohl M.G., Gladman D.D., Wu J.J., Mehta N.N., Finlay A.Y., Gottlieb A.B. (2016). Psoriasis. Nat. Rev. Dis. Primers.

[6] Torres F., das Graças M., Melo M., Tosti A. (2009). Management of contact dermatitis due to nickel allergy: An update. Clin. Cosmet. Investig. Dermatol..

[7] Chen H., Liu G., Huang N., Li W., Dong X., Zhu R. (2016). Incidence of allergic contact sensitization in central Chinese subjects with chronic urticaria. An. Bras. Dermatol..

[8] Bjerre R.D., Bandier J., Skov L., Engstrand L., Johansen J.D. (2017). The role of the skin microbiome in atopic dermatitis: A systematic review. Br. J. Dermatol..

[9] Dréno B. (2017). What is new in the pathophysiology of acne, an overview. J. Eur. Acad. Dermatol. Venereol..

[10] Richmond J.M., Harris J.E. (2014). Immunology and skin in health and disease. Cold Spring Harb. Perspect. Med..

[11] Bickers D.R., Athar M. (2006). Oxidative stress in the pathogenesis of skin disease. J. Investig. Dermatol..

[12] Ingram S.L., Diotallevi M. (2017). Reactive oxygen species: Rapid fire inflammation. Biochemist.

[13] Briganti S., Picardo M. (2003). Antioxidant activity, lipid peroxidation and skin diseases. What’s new. J. Eur. Acad. Dermatol. Venereol..

[14] Reuter S., Gupta S.C., Chaturvedi M.M., Aggarwal B.B. (2010). Oxidative stress, inflammation, and cancer: How are they linked?. Free Radic. Biol. Med..

[15] Ferlazzo N., Cirmi S., Calapai G., Ventura-Spagnolo E., Gangemi S., Navarra M. (2016). Anti-Inflammatory Activity of Citrus bergamia Derivatives: Where Do We Stand?. Molecules.

[16] Conte R., Marturano V., Peluso G., Calarco A., Cerruti P. (2017). Recent Advances in Nanoparticle-Mediated Delivery of Anti-Inflammatory Phytocompounds. Int. J. Mol. Sci..

[17] Puglia C., Lauro M.R., Offerta A., Crascì L., Micicchè L., Panico A.M., Bonina F., Puglisi G. (2017). Nanostructured Lipid Carriers (NLC) as Vehicles for Topical Administration of Sesamol: In Vitro Percutaneous Absorption Study and Evaluation of Antioxidant Activity. Planta Med..

[18] Daneshmand S., Jaafari M.R., Movaffagh J., Malaekeh-Nikouei B., Iranshahi M., Seyedian Moghaddam A., Tayarani Najaran Z., Golmohammadzadeh S. (2018). Preparation, characterization, and optimization of auraptene-loaded solid lipid nanoparticles as a natural anti-inflammatory agent: In vivo and in vitro evaluations. Colloids Surf. B Biointerfaces.

[19] Castro G.A., Oliveira C.A., Mahecha G.A., Ferreira L.A. (2011). Comedolytic effect and reduced skin irritation of a new formulation of all-trans retinoic acid-loaded solid lipid nanoparticles for topical treatment of acne. Arch. Dermatol. Res..

[20] Guo C.-Y., Yang C.-F., Li Q.L., Tan Q., Xi Y.-W., Liu W.-N., Zhai G.-X. (2012). Development of a quercetin-loaded nanostructured lipid carrier formulation for topical delivery. Int. J. Pharm..

[21] Kakkar V., Kaur I.P., Kaur A.P., Saini K., Singh K.K. (2018). Topical delivery of tetrahydrocurcumin lipid nanoparticles effectively inhibits skin inflammation: In vitro and in vivo study. Drug Dev. Ind. Pharm..

[22] Schäfer-Korting M., Mehnert W., Korting H.C. (2007). Lipid nanoparticles for improved topical application of drugs for skin diseases. Adv. Drug Deliv. Rev..

[23] Küchler S., Radowski M.R., Blaschke T., Dathe M., Plendl J., Haag R., Schäfer-Korting M., Kramer K.D. (2009). Nanoparticles for skin penetration enhancement—A comparison of a dendritic core-multishell-nanotransporter and solid lipid nanoparticles. Eur. J. Pharm. Biopharm..

[24] Bikkad M.L., Nathani A.H., Mandlik S.K., Shrotriya S.N., Ranpise N.S. (2014). Halobetasol propionate-loaded solid lipid nanoparticles (SLN) for skin targeting by topical delivery. J. Liposome Res..

[25] Pang Z., Han C. (2014). Review on Transdermal Drug Delivery Systems. J. Pharm. Drug Dev..

[26] Shrotriya S., Ranpise N., Satpute P., Vidhate B. (2018). Skin targeting of curcumin solid lipid nanoparticles-engrossed topical gel for the treatment of pigmentation and irritant contact dermatitis. Artif. Cells Nanomed. Biotechnol..

[27] Wissing S.A., Müller R.H. (2003). The influence of solid lipid nanoparticles on skin hydration and viscoelasticity—In vivo study. Eur. J. Pharm. Biopharm..

[28] Khalid K., Tan X., Zaid H.F.M., Tao Y., Chew C.L., Chu D.T., Lam M.K., Ho Y.C., Lim J.W., Wei L.C. (2020). Advanced in developmental organic and inorganic nanomaterial: A review. Bioengineered.

[29] Palmer R.E. (2012). Nanobiotechnology: Inorganic Nanoparticles vs Organic Nanoparticles.

[30] Zhao Z., Liu T., Zhu S., Pi J., Guo P., Qi D., Liu Z., Li N. (2021). Natural medicine combined with nanobased topical delivery systems: A new strategy to treat psoriasis. Drug Deliv. Transl. Res..

[31] Szulc-Musioł B., Sarecka-Hujar B. (2021). The Use of Micro- and Nanocarriers for Resveratrol Delivery into and across the Skin in Different Skin Diseases—A Literature Review. Pharmaceutics.

[32] Biswasroy P., Pradhan D., Kar B., Ghosh G., Rath G. (2021). Recent Advancement in Topical Nanocarriers for the Treatment of Psoriasis. AAPS PharmSciTech.

[33] Dhiman N., Awasthi R., Sharma B., Kharkwal H., Kulkarni G.T. (2021). Lipid Nanoparticles as Carriers for Bioactive Delivery. Front. Chem..

[34] Küchler S., Herrmann W., Panek-Minkin G., Blaschke T., Zoschke C., Kramer K.D., Bittl R., Schäfer-Korting M. (2010). SLN for topical application in skin diseases—Characterization of drug-carrier and carrier-target interactions. Int. J. Pharm..

[35] Barua S., Mitragotri S. (2014). Challenges associated with Penetration of Nanoparticles across Cell and Tissue Barriers: A Review of Current Status and Future Prospects. Nano Today.

[36] Lai-Cheong J.E., McGrath J.A. (2009). Structure and function of skin, hair, and nails. Medicine.

[37] Young C.N., Koepke J.I., Terlecky L.J., Forquin M.S., Boyd Savoy L., Terlecky S.R. (2008). Reactive oxygen species in tumor necrosis factor-alpha-activated primary human keratinocytes: Implications for psoriasis and inflammatory skin disease. J. Investig. Dermatol..

[38] Wickett R.R., Visscher M.O. (2006). Structure and function of the epidermal barrier. Am. J. Infect. Control..

[39] Barry B.W. (2001). Novel mechanisms and devices to enable successful transdermal drug delivery. Eur. J. Pharm. Sci..

[40] Bouwstra J.A., Honeywell-Nguyen P.L., Gooris G.S., Ponec M. (2003). Structure of the skin barrier and its modulation by vesicular formulations. Prog. Lipid Res..

[41] Cevc G. (2004). Lipid vesicles and other colloids as drug carriers on the skin. Adv. Drug Deliv. Rev..

[42] Gu Y., Yang M., Tang X., Wang T., Yang D., Zhai G., Liu J. (2018). Lipid nanoparticles loading triptolide for transdermal delivery: Mechanisms of penetration enhancement and transport properties. J. Nanobiotechnology.

[43] Khurana S., Jain N.K., Bedi P.M. (2013). Development and characterization of a novel controlled release drug delivery system based on nanostructured lipid carriers’ gel for meloxicam. Life Sci..

[44] Arda O., Göksügür N., Tüzün Y. (2014). Basic histological structure and functions of facial skin. Clin. Dermatol..

[45] Murphree R.W. (2017). Impairments in Skin Integrity. Nurs. Clin. N. Am..

[46] Gould J. (2018). Superpowered skin. Nature.

[47] Kabashima K., Honda T., Ginhoux F., Egawa G. (2019). The immunological anatomy of the skin. Nat. Rev. Immunol..

[48] Nguyen A.V., Soulika A.M. (2019). The dynamics of the skin’s immune system. Int. J. Mol. Sci..

[49] Johansson J.A., Headon D.J. (2014). Regionalisation of the skin. Semin. Cell Dev. Biol..

[50] Prausnitz M.R., Mitragotri S., Langer R. (2004). Current status and future potential of transdermal drug delivery. Nat. Rev. Drug Discov..

[51] Pando D., Matos M., Gutiérrez G., Pazos C. (2015). Formulation of resveratrol entrapped niosomes for topical use. Colloids Surf. B Biointerfaces.

[52] Dawid-Pać R. (2013). Medicinal plants used in treatment of inflammatory skin diseases. Postepy Dermatol. Alergol..

[53] Stupin V., Manturova N., Silina E., Litvitskiy P., Vasin V., Artyushkova E., Inanov A., Gladchenko M., Aliev S. (2020). The effect of inflammation on the healing process of acute skin wounds under the treatment of wounds with injections in rats. J. Exp. Pharmacol..

[54] Yuan H., Ma Q., Ye L., Piao G. (2016). The Traditional Medicine and Modern Medicine from Natural Products. Molecules.

[55] Newman D.J., Cragg G.M., Snader K.M. (2003). Natural products as sources of new drugs over the Period 1981–2002. J. Nat. Prod..

[56] Kabir M.G., Rahman M.M., Ahmed N.U., Fakruddin M., Islam S., Mazumdar R.M. (2014). Antioxidant, antimicrobial, toxicity, and analgesic properties of ethanol extract of Solena amplexicaulis root. Biol. Res..

[57] Azab A., Nassar A., Azab A.N. (2016). Anti-Inflammatory Activity of Natural Products. Molecules.

[58] Arulselvan P., Fard M.T., Tan W.S., Gothai S., Fakurazi S., Norhaizan M.E., Kumar S.S. (2016). Role of Antioxidants and Natural Products in Inflammation. Oxid. Med. Cell. Longev..

[59] Lin T.K., Zhong L., Santiago J.L. (2017). Anti-Inflammatory, and Skin Barrier Repair Effects of Topical Application of Some Plant Oils. Int. J. Mol. Sci..

[60] Prasad S., Phromnoi K., Yadav V.R., Chaturvedi M.M., Aggarwal B.B. (2010). Targeting inflammatory pathways by flavonoids for prevention and treatment of cancer. Planta Med..

[61] Cordenonsi L.M., Santer A., Sponchiado R.M., Wingert N.R., Raffin R.P., Schapoval E.E.S. (2019). Amazonia Products in Novel Lipid Nanoparticles for Fucoxanthin Encapsulation. AAPS PharmSciTech.

[62] Heo S.-J., Yoon W.J., Kim K.N., Ahn G.N., Kang S.M., Kang D.H., Affan A., Oh C., Jung W.K., Jeon Y.J. (2010). Evaluation of anti-inflammatory effect of fucoxanthin isolated from brown algae in lipopolysaccharide-stimulated RAW 264.7 macrophages. Food Chem. Toxicol..

[63] Oh J., Kim J.H., Park J.G., Yi Y.S., Park K.W., Rho H.S., Lee M.S., Yoo J.W., Kang S.H., Hong Y.D. (2013). Radical scavenging activity-based and AP-1-targeted anti-inflammatory effects of lutein in macrophage-like and skin keratinocytic cells. Mediat. Inflamm..

[64] Chung R.W.S., Leanderson P., Lundberg A.K., Jonasson L. (2017). Lutein exerts anti-inflammatory effects in patients with coronary artery disease. Atherosclerosis.

[65] Hamidzadeh K., Christensen S.M., Dalby E., Chandrasekaran P., Mosser D.M. (2017). Macrophages and the Recovery from Acute and Chronic Inflammation. Annu. Rev. Physiol..

[66] Landrier J.F., Tourniaire F., Fenni S., Desmarchelier C., Borel P., Rao A.V., Young G.L., Rao L.G. (2018). Tomatoes and lycopene: Inflammatory modulator effects. Lycopene and Tomatoes in Human Nutrition and Health.

[67] Chen J., Song Y., Zhang L. (2013). Effect of lycopene supplementation on oxidative stress: An exploratory systematic review and meta-analysis of randomized controlled trials. J. Med. Food.

[68] Palozza P., Parrone N., Catalano A., Simone R. (2010). Tomato Lycopene, and Inflammatory Cascade: Basic Interactions and Clinical Implications. Curr. Med. Chem..

[69] Chang Y.C., Tsai M.H., Sheu W.H., Hsieh S.C., Chiang A.N. (2013). The therapeutic potential and mechanisms of action of quercetin in relation to lipopolysaccharide-induced sepsis in vitro and in vivo. PLoS ONE.

[70] Gunawardena D., Govindaraghavan S., Münch G., Watson R.R., Preedy V.R., Zibadi S. (2014). Anti-Inflammatory Properties of Cinnamon Polyphenols and their Monomeric Precursors. Polyphenols in Human Health and Disease.

[71] Nijveldt R.J., van Nood E., van Hoorn D.E., Boelens P.G., van Norren K., van Leeuwen P.A. (2001). Flavonoids: A review of probable mechanisms of action and potential applications. Am. J. Clin. Nutr..

[72] PubChem (2019). Quercetin. https://pubchem.ncbi.nlm.nih.gov/compound/quercetin#section=Pharmacology.

[73] Ha T.K.K., Bennett P.N., Brown M.J., Sharma P. (2012). Drugs, and the skin. Clinical Pharmacology.

[74] Li X., Yu C., Hu Y., Xia X., Liao Y., Zhang J., Chen H., Lu W., Zhou W., Song Z. (2018). New Application of Psoralen and Angelicin on Periodontitis with Anti-bacterial, Anti-inflammatory, and Osteogenesis Effects. Front. Cell. Infect. Microbiol..

[75] Fadus M.C., Lau C., Bikhchandani J., Lynch H.T. (2016). Curcumin: An age-old anti-inflammatory and anti-neoplastic agent. J. Tradit. Complement. Med..

[76] Hewlings S.J., Kalman D.S. (2017). Curcumin: A Review of Its’ Effects on Human Health. Foods.

[77] PubChem (2019). Curcumin. https://pubchem.ncbi.nlm.nih.gov/compound/curcumin.

[78] Das S., Das D.K. (2007). Anti-inflammatory responses of resveratrol. Inflamm. Allergy Drug Targets.

[79] Švajger U., Jeras M. (2012). Anti-inflammatory effects of resveratrol and its potential use in therapy of immune-mediated diseases. Int. Rev. Immunol..

[80] Zhong L.M., Zong Y., Sun L., Guo J.Z., Zhang W., He Y., Song R., Wang W.M., Xiao C.J., Lu D. (2012). Resveratrol inhibits inflammatory responses via the mammalian target of rapamycin signaling pathway in cultured LPS-stimulated microglial cells. PLoS ONE..

[81] Poulsen M.M., Fjeldborg K., Ornstrup M.J., Kjær T.N., Nøhr M.K., Pedersen S.B. (2015). Resveratrol and inflammation: Challenges in translating pre-clinical findings to improved patient outcomes. Biochim. Et Biophys. Acta (BBA)-Mol. Basis Dis..

[82] Coutinho D.S., Pacheco M.T., Frozza R.L., Bernardi A. (2018). Anti-Inflammatory Effects of Resveratrol: Mechanistic Insights. Int. J. Mol. Sci..

[83] BaGen H., Liu X., Han J. (2018). The anti-inflammation effects of resveratrol for patients after oral implantology. Biomed. Res..

[84] Chu P.Y., Hsu D.Z., Hsu P.Y., Liu M.Y. (2010). Sesamol down-regulates the lipopolysaccharide-induced inflammatory response by inhibiting nuclear factor-kappa B activation. Innate Immun..

[85] Yashaswini P.S., Rao A.G., Singh S.A. (2017). Inhibition of lipoxygenase by sesamol corroborates its potential anti-inflammatory activity. Int. J. Biol. Macromol..

[86] Sá R.C.S., Andrade L.N., de Sousa D.P. (2013). A review on anti-inflammatory activity of monoterpenes. Molecules.

[87] Garcia S.C., Lopes L.S., Schott K.L., Beck S.T., Pomblum V.J. (2004). Ciclosporina A and tacrolimus: Uma revisão. J. Bras. Patol. Med. Lab..

[88] Lemster B.H., Carroll P.B., Rilo H.R., Johnson N., Nikaein A., Thomson A.W. (1995). IL-8/IL-8 receptor expression in psoriasis and the response to systemic tacrolimus (FK506) therapy. Clin. Exp. Immunol..

[89] Emal D., Rampanelli E., Claessen N., Bemelman F.J., Leemans J.C., Florquin S., Dessing M.C. (2019). Calcineurin inhibitor Tacrolimus impairs host immune response against urinary tract infection. Sci. Rep..

[90] Mu H., Holm R. (2018). Solid lipid nanocarriers in drug delivery: Characterization and design. Expert Opin. Drug Deliv..

[91] Pivetta T.P., Simões S., Araújo M.M., Carvalho T., Arruda C., Marcato P.D. (2018). Development of nanoparticles from natural lipids for topical delivery of thymol: Investigation of its anti-inflammatory properties. Colloids Surf. B Biointerfaces.

[92] Puglia C., Bonina F. (2012). Lipid nanoparticles as novel delivery systems for cosmetics and dermal pharmaceuticals. Expert Opin. Drug Deliv..

[93] Souto E.B., Wissing S.A., Barbosa C.M., Müller R.H. (2004). Evaluation of the physical stability of SLN and NLC before and after incorporation into hydrogel formulations. Eur. J. Pharm. Biopharm..

[94] Sanap G.S., Mohanta G.P. (2014). Investigation of the factors influencing the incorporation of miconazole in SNL and NLC dispersion. IAJPS.

[95] Müller R.H., Lucks J.S. (1996). Inventors. Arzneistoffträger aus Festen Lipidteilchen, Feste Lipidnanosphären (SLN). European Patent.

[96] Müller R.H., Radtke M., Wissing S.A. (2002). Solid lipid nanoparticles (SLN) and nanostructured lipid carriers (NLC) in cosmetic and dermatological preparations. Adv. Drug Deliv. Rev..

[97] Kammari R., Das N.G., Das S.K., Mitra A., Cholkar K., Mandal A. (2017). Nanoparticulate Systems for Therapeutic and Diagnostic Applications. Emerging Nanotechnologies for Diagnostics, Drug Delivery and Medical Devices.

[98] Zhai Y., Zhai G. (2014). Advances in lipid-based colloid systems as drug carrier for topic delivery. J. Control. Release.

[99] Müller R.H., Petersen R.D., Hommoss A., Pardeike J. (2007). Nanostructured lipid carriers (NLC) in cosmetic dermal products. Adv. Drug Deliv. Rev..

[100] Ram D.T., Debnath S., Babu M.N., Nath T.C., Thejeswi B. (2012). A review on solid lipid nanoparticles. RJPT.

[101] Souto E.B., Müller R.H. (2008). Cosmetic features, and applications of lipid nanoparticles (SLN, NLC). Int. J. Cosmet. Sci..

[102] Jensen L.B., Petersson K., Nielsen H.M. (2011). In vitro penetration properties of solid lipid nanoparticles in intact and barrier-impaired skin. Eur. J Pharm. Biopharm..

[103] Nirbhavane P., Sharma G., Singh B., Khuller G.K., Goni V.G., Patil A.B., Katare O.P. (2018). Preclinical Explorative Assessment of Celecoxib-Based Biocompatible Lipidic Nanocarriers for the Management of CFA-Induced Rheumatoid Arthritis in Wistar Rats. AAPS PharmSciTech.

[104] Rocha V., Marques C., Figueiredo J.L., Gaio A.R., Costa P.C., Sousa Lobo J.M., Almeida I.F. (2017). In vitro cytotoxicity evaluation of resveratrol-loaded nanoparticles: Focus on the challenges of in vitro methodologies. Food Chem. Toxicol..

[105] Wissing S., Lippacher A., Müller R. (2001). Investigations on the occlusive properties of solid lipid nanoparticles (SLN). J. Cosmet. Sci..

[106] Charcosset C., El-Harati A., Fessi H. (2005). Preparation of solid lipid nanoparticles using a membrane contactor. J. Control. Release.

[107] Fang J.Y., Fang C.L., Liu C.H., Su Y.H. (2008). Lipid nanoparticles as vehicles for topical psoralen delivery: Solid lipid nanoparticles (SLN) versus nanostructured lipid carriers (NLC). Eur. J. Pharm. Biopharm..

[108] Montenegro L., Panico A.M., Santagati L.M., Siciliano E.A., Intagliata S., Modica M.N. (2018). Solid Lipid Nanoparticles Loading Idebenone Ester with Pyroglutamic Acid: In Vitro Antioxidant Activity and In Vivo Topical Efficacy. Nanomaterials.

[109] Pallerla S.M., Prabhakar B.R. (2013). A Review on Solid Lipid Nanoparticles. Int. J. Pharm. Sci. Rev. Res..

[110] Rostamkalaei S.S., Akbari J., Saeedi M., Morteza-Semnani K., Nokhodchi A. (2019). Topical gel of Metformin solid lipid nanoparticles: A hopeful promise as a dermal delivery system. Colloids Surf. B Biointerfaces.

[111] Ruktanonchai U., Bejrapha P., Sakulkhu U., Opanasopit P., Bunyapraphatsara N., Junyaprasert V., Puttipipatkhachorn S. (2009). Physicochemical characteristics, cytotoxicity, and antioxidant activity of three lipid nanoparticulate formulations of alpha-lipoic acid. AAPS PharmSciTech.

[112] Wang J.J., Liu K.S., Sung K.C., Tsai C.Y., Fang J.Y. (2009). Lipid nanoparticles with different oil/fatty ester ratios as carriers of buprenorphine and its prodrugs for injection. Eur. J. Pharm. Sci..

[113] Soldati P.P., Polonini H.C., Paes C.Q., Restrepob J.A.S., Creczynksi-Pasa T.B., Chaves M.G.A.M., Brandão M.A.F., Pittella F., Raposo N.R.B. (2018). Controlled release of resveratrol from lipid nanoparticles improves antioxidant effect. IFAC-PapersOnLine.

[114] Mehnert W., Mäder K. (2001). Solid lipid nanoparticles: Production, characterization, and applications. Adv. Drug Deliv. Rev..

[115] Jenning V., Lippacher A., Gohla S.H. (2002). Medium scale production of solid lipid nanoparticles (SLN) by high pressure homogenization. J. Microencapsul..

[116] Zielińska A., Martins-Gomes C., Ferreira N.R., Silva A.M., Nowak I., Souto E.B. (2018). Anti-inflammatory and anti-cancer activity of citral: Optimization of citral-loaded solid lipid nanoparticles (SLN) using experimental factorial design and LUMiSizer^®^. Int. J. Pharm..

[117] Pizzol C.D., Filippin-Monteiro F.B., Restrepo J.A., Pittella F., Silva A.H., Alves de Souza P., Machado de Campos A., Creczynski-Pasa T.B. (2014). Influence of surfactant and lipid type on the physicochemical properties and biocompatibility of solid lipid nanoparticles. Int. J. Environ. Res. Public Health.

[118] Rigon R.B., Gonçalez M.L., Severino P., Alves D.A., Santana M.H.A., Souto E.B., Chorilli M. (2018). Solid lipid nanoparticles optimized by 22 factorial design for skin administration: Cytotoxicity in NIH3T3 fibroblasts. Colloids Surf. B Biointerfaces.

[119] Altube M.J., Cutro A., Bakas L., Morilla M.J., Disalvo E.A., Romero E.L. (2017). Nebulizing novel multifunctional nanovesicles: The impact of macrophage-targeted-pH-sensitive archaeosomes on a pulmonary surfactant. J. Mater. Chem. B.

[120] Higa L.H., Jerez H.E., de Farias M.A., Portugal R.V., Romero E.L., Morilla M.J. (2017). Ultra-small solid archaeolipid nanoparticles for active targeting to macrophages of the inflamed mucosa. Nanomedicine.

[121] Müller R.H., Mäder K., Gohla S. (2000). Solid lipid nanoparticles (SLN) for controlled drug delivery—A review of the state of the art. Eur. J. Pharm. Biopharm..

[122] Wong H.L., Bendayan R., Rauth A.M., Li Y., Wu X.Y. (2007). Chemotherapy with anticancer drugs encapsulated in solid lipid nanoparticles. Adv. Drug Deliv. Rev..

[123] Zamarioli C.M., Martins R.M., Carvalho E.C., Freitas L.A.P. (2015). Nanoparticles containing curcuminoids (Curcuma longa): Development of topical delivery formulation. Rev. Bras. Farmacogn..

[124] Mishra V., Bansal K.K., Verma A., Yadav N., Thakur S., Sudhakar K., Rosenholm J.M. (2018). Solid lipid nanoparticles: Emerging colloidal nano drug delivery system. Pharmaceutics.

[125] Garud A., Singh D., Garud N. (2012). Solid Lipid Nanoparticles (SLN): Method, Characterization and Applications. Int. Curr. Pharm. J..

[126] Maa Y.F., Hsu C.C. (1999). Performance of sonication and microfluidization for liquid-liquid emulsification. Pharm. Dev. Technol..

[127] Hielscher T. (2005). Ultrasonic Production of Nano-Size Dispersions and Emulsions. Proceedings of the 5th ENS@T Scientific Meeting, Paris, France, 9–10 December 2005.

[128] Cavalli R., Caputo O., Carlotti M.E., Trotta M., Scarnecchia C., Gasco M.R. (1997). Sterilization and freeze-drying of drug-free and drug-loaded solid lipid nanoparticles. Int. J. Pharm..

[129] Munin A., Edwards-Lévy F. (2011). Encapsulation of Natural Polyphenolic Compounds; A Review. Pharmaceutics.

[130] Leskošek-Čukalović I.J., Despotović S.M., Nedović V.A., Nikšić M.P. (2009). Medicinal mushroom Ganoderma lucidum in the production of special beer types. Zb. Matice Srp. Prir. Nauk..

[131] Mura P., Maestrelli F., D’Ambrosio M., Luceri C., Cirri M. (2021). Evaluation and comparison of Solid Lipid Nanoparticles (SLNs) and Nanostructured Lipid Carriers (NLCs) as vectors to develop hydrochlorothiazide effective and safe pediatric oral liquid formulations. Pharmaceutics.

[132] Oliveira M.S., Goulart G.C.A., Ferreira L.A.M., Carneiro G. (2017). Hydrophobic ion pairing as a strategy to improve drug encapsulation into lipid nanocarriers for the cancer treatment. Expert Opin. Drug Deliv..

[133] Zorzi G.K., Carvalho E.L.S., Poser G.L., Teixeira H.F. (2015). On the use of nanotechnology-based strategies for association of complex matrices from plants extracts. Rev. Bras. Farmacogn..

[134] Subramaniam B., Siddik Z.H., Nagoor N.H. (2020). Optimization of nanostructured lipid carriers: Understanding the types, designs, and parameters in the process of formulations. J. Nanopart. Res..

[135] Pacheco-Fernández I., Pino V., Poole C. (2019). Extraction with ionic liquids-organic compounds. Liquid-Phase Extraction.

[136] Radünz M., Hackbart H.C.S., Camargo T.M., Nunes C.F.P., de Barros F.A.P., Dal Magro J.D., Sanchez Filho P.J., Gandra E.A., Radünz A.L., Zavareze E.R. (2020). Antimicrobial potential of spray drying encapsulated thyme (*Thymus vulgaris*) essential oil on the conservation of hamburger-like meat products. Intern. J. Food Microbiol..

[137] Watkins R., Wu L., Zhang C., Davis R.M., Xu B. (2015). Natural product-based nanomedicine: Recent advances and issues. Int. J. Nanomed..

[138] Kim S.T., Jang D.J., Kim J.H., Park J.Y., Lim J.S., Lee S.Y., Lee K.M., Lim S.J., Kim C.K. (2009). Topical administration of cyclosporin A in a solid lipid nanoparticle formulation. Pharmazie.

[139] Essaghraoui A., Belfkira A., Hamdaoui B., Nunes C., Lima S.A.C., Reis S. (2019). Improved Dermal Delivery of Cyclosporine A Loaded in Solid Lipid Nanoparticles. Nanomaterials.

[140] Silva M.I., Barbosa A.I., Costa Lima S.A., Costa P., Torres T., Reis S. (2020). Freeze-Dried Softisan^®^ 649-Based Lipid Nanoparticles for Enhanced Skin Delivery of Cyclosporine, A. Nanomaterials.

[141] Trombino S., Servidio C., Laganà A.S., Conforti F., Marrelli M., Cassano R. (2020). Viscosified Solid Lipidic Nanoparticles Based on Naringenin and Linolenic Acid for the Release of Cyclosporine A on the Skin. Molecules.

[142] Arora R., Katiyar S.S., Kushwah V., Jain S. (2017). Solid lipid nanoparticles and nanostructured lipid carrier-based nanotherapeutics in treatment of psoriasis: A comparative study. Expert Opin. Drug Deliv..

[143] Caon T., Mazzarino L., Simões C.M., Senna E.L., Silva M.A. (2017). Lipid- and Polymer-Based Nanostructures for Cutaneous Delivery of Curcumin. AAPS PharmSciTech.

[144] Iriventi P., Gupta N.V. (2020). Topical delivery of curcumin and caffeine mixture-loaded nanostructured lipid carriers for effective treatment of psoriasis. Pharmacogn. Mag..

[145] Mitri K., Shegokar R., Gohla S., Anselmi C., Müller R.H. (2011). Lipid nanocarriers for dermal delivery of lutein: Preparation, characterization, stability, and performance. Int. J. Pharm..

[146] Okonogi S., Riangjanapatee P. (2015). Physicochemical characterization of lycopene-loaded nanostructured lipid carrier formulations for topical administration. Int. J. Pharm..

[147] Bose S., Michniak-Kohn B. (2013). Preparation and characterization of lipid based nanosystems for topical delivery of quercetin. Eur. J. Pharm. Sci..

[148] Sun R., Zhao G., Ni S., Xia Q. (2014). Lipid based nanocarriers with different lipid compositions for topical delivery of resveratrol: Comparative analysis of characteristics and performance. J. Drug Deliv. Sci. Technol..

[149] Viegas J.S.R., Praca F.G., Caron A.L., Suzuki I., Silvestrini A.V.P., Medina W.S.G., Ciampo J.O.D., Kravicz M., Bentley M.V.L.B. (2020). Nanostructured lipid carrier co-delivering tacrolimus and TNF-α siRNA as an innovate approach to psoriasis. Drug Deliv. Transl. Res..

[150] Gárcia-Pinel B., Porras-Alcalá C., Ortega-Rodríguez A., Sarabia F., Prados J., Melguizo C., López-Romero J.M. (2019). Lipid-Based Nanoparticles: Application and Recent Advances in Cancer Treatment. Nanomaterials.

[151] Thurnham D.I., Northrop-Clewes C.A. (2016). Inflammation and biomarkers of micronutrient status. Curr. Opin. Clin. Nutr. Metab. Care.

[152] Ford E.S., Liu S., Mannino D.M., Giles W.H., Smith S.J. (2003). C-reactive protein concentration and concentrations of blood vitamins, carotenoids, and selenium among United States adults. Eur. J. Clin. Nutr..

[153] Lacatusu I., Badea G., Popescu M., Bordei N., Istrati D., Moldovan L., Seciu A.M., Panteli M.I., Rasit I., Badea N. (2017). Marigold extract, azelaic acid and black caraway oil into lipid nanocarriers provides a strong anti-inflammatory effect in vivo. Ind. Crop. Prod..

[154] Faiyazuddin M., Akhtar N., Akhter J., Suri S., Shakeel F., Shafiq S., Mustafa G. (2010). Production, characterization, in vitro and ex vivo studies of babchi oil-encapsulated nanostructured solid lipid carriers produced by a hot aqueous titration method. Pharmazie.

[155] Varman R.M., Singh S. (2012). Investigation of effects of terpene skin penetration enhancers on stability and biological activity of lysozyme. AAPS PharmSciTech.

[156] Gallily R., Yekhtin Z., Hanuš L.O. (2018). The Anti-Inflammatory Properties of Terpenoids from Cannabis. Cannabis Cannabinoid Res..

[157] Afra B., Mohammadi M., Soleimani M., Mahjub R. (2020). Preparation, Statistical Optimization, In Vitro Characterization, and In Vivo Pharmacological Evaluation of Solid Lipid Nanoparticles Encapsulating Propolis Flavonoids: A Novel Treatment for Skin Edema. Drug Dev. Ind. Pharm..

